# Blood Reference Intervals for Preterm Low-Birth-Weight Infants: A Multicenter Cohort Study in Japan

**DOI:** 10.1371/journal.pone.0161439

**Published:** 2016-08-23

**Authors:** Masayuki Ochiai, Yuki Matsushita, Hirosuke Inoue, Takeshi Kusuda, Dongchon Kang, Kiyoshi Ichihara, Naoki Nakashima, Kenji Ihara, Shouichi Ohga, Toshiro Hara, Kyushu University High-Risk Neonatal Clinical Research Network, Japan

**Affiliations:** 1 Department of Pediatrics, Graduate School of Medical Sciences, Kyushu University, Fukuoka, Japan; 2 Comprehensive Maternity and Perinatal Care Center, Kyushu University Hospital, Fukuoka, Japan; 3 Department of Clinical Chemistry and Laboratory Medicine, Kyushu University, Fukuoka, Japan; 4 Department of Clinical Laboratory Sciences, Yamaguchi University, Ube, Japan; 5 Medical Information Center, Kyushu University Hospital, Fukuoka, Japan; 6 Department of Perinatal and Pediatric Medicine, Graduate School of Medical Sciences, Kyushu University, Fukuoka, Japan; 7 Department of Pediatrics, Faculty of Medicine and Health Sciences, Yamaguchi University, Ube, Japan; University of Cape Town, SOUTH AFRICA

## Abstract

Preterm low-birth-weight infants remain difficult to manage based on adequate laboratory tests. The aim of this study was to establish blood reference intervals (RIs) in those newborns who were admitted to and survived in the neonatal intensive care unit (NICU). A multicenter prospective study was conducted among all infants admitted to 11 affiliated NICUs from 2010 to 2013. The clinical information and laboratory data were registered in a network database designed for this study. The RIs for 26 items were derived using the parametric method after applying the latent abnormal values exclusion method. The influence of birth weight (BW) and gestational age (GA) on the test results was expressed in terms of the standard deviation ratio (SDR), as SDR_BW_ and SDR_GA_, respectively. A total of 3189 infants were admitted during the study period; 246 were excluded due to a lack of blood sampling data, and 234 were excluded for chromosomal abnormalities (n = 108), congenital anomalies requiring treatment with surgical procedures (n = 76), and death or transfer to another hospital (n = 50). As a result, 2709 infants were enrolled in this study. Both the SDR_GA_ and SDR_BW_ were above 0.4 in the test results for total protein (TP), albumin (ALB), alanine aminotransferase (ALT), and red blood cells (RBC); their values increased in proportion to the BW and GA. We derived 26 blood RIs for infants who were admitted to NICUs. These RIs should help in the performance of proper clinical assessments and research in the field of perinatal-neonatal medicine.

## Introduction

The prognosis of preterm low-birth-weight infants has improved dramatically with advances in perinatal medicine. The Neonatal Research Network of Japan revealed that more than 80% of infants delivered at 24 weeks of gestational age (GA) survived in neonatal intensive care units (NICUs) [1], and the survival rates of infants born at GA 22 and 23 weeks were also improved compared with those in previous studies [2]. However, the treatment of these vulnerable newborns and the associated clinical research remain great challenges. It is essential to properly assess these infants based on adequate physiological data [3] as well as laboratory tests.

Due to the nature of the physiological growth and development of infants and children, many efforts have been made to establish pediatric reference intervals (RIs) for routinely-measured laboratory parameters[4] [5]. Christensen et al. published RIs for the complete blood cell (CBC) counts in term and preterm infants using the large database of a health care system [6] [7] [8] [9]. The Canadian Laboratory Initiative on Pediatric Reference Interval Database established RIs for blood chemistry data in healthy and multiethnic child populations [10] [5]. In contrast, few studies have been published regarding the RIs of blood chemistry elements for preterm low-birth-weight infants[11] [12] [13].

The “Kyushu University High-Risk Neonatal Clinical Research Network” Project is a collaborative study conducted among NICUs across the northern part of Kyushu Island in Japan from April 2010 to March 2013. For this project, a prospective observational study was performed in the 11 affiliated hospitals in order to establish blood RIs in preterm low-birth-weight infants within 24 h after birth who were admitted to multiple perinatal care centers and who survived until hospital discharge.

## Materials and Methods

### Multicenter Prospective Study

All infants admitted to any of the 11 affiliated NICUs on the first day of life were enrolled in this study, with the following exclusion criteria: chromosomal abnormalities, congenital anomalies requiring surgical procedures during the neonatal period, and death or transfer to other hospitals prior to discharge. Data acquisition was carried out using a web-based electronic medical software program that stores laboratory data and clinical information (Hitachi Solutions, Ltd., Tokyo, Japan). The data were classified into three subgroups based on either by BW or GA according to the classification of the World Health Organization (WHO) for neonates. The study protocol was approved by the Institutional Review Board (#22–131; Kyushu University Hospital) at each institution and registered as a prospective observational study with the University Hospital Medical Information Network clinical trial registration system in Japan (UMIN000008763) in April 2010. Written informed consent was obtained from all of the caretakers of the patients prior to their enrollment in this study.

### Target Test Items and Standardization of Measurements

The Japan Society of Clinical Chemistry established common RIs for use nationwide in Japan for 40 commonly tested laboratory tests [14]. Annual external quality controls have been done for the major analytes among the affiliated medical facilities in the northern region of Kyushu Island[15], and we confirmed the standardized status of all the assays[16]. The CBC count and differential white blood cell counts were measured using automated Beckman Coulter Hematology Analyzers (Beckman Coulter Inc., FL, USA). The following 16 biochemical and 10 hematological test items were chosen as analytes: total protein (TP), albumin (ALB), blood urea nitrogen (BUN), creatinine (CRE), total bilirubin (T-BIL), direct bilirubin (D-BIL), sodium (Na), potassium (K), chlorine (CL), calcium (Ca), C-reactive protein (CRP), aspartate aminotransferase (AST), alanine aminotransferase (ALT), lactate dehydrogenase (LDH), alkaline phosphatase (ALP), creatine kinase (CK), white blood cells (WBC), red blood cells (RBC), hemoglobin (HGB), hematocrit (HCT), platelets (PLT), neutrophils (NEUT), lymphocytes (LYMP), monocytes (MONO), eosinophils (EOS) and basophils (BASO).

### Statistical Analysis

The following items, which may be affected by a pathological state, were excluded depending on the international classification of diseases-10 (ICD-10) code: LDH, AST, and CK in infants with a disease code of P20-29; respiratory and cardiovascular disorders specific to the perinatal period and CRP in infants with P35-39; infections specific to the perinatal period, K, and T-BIL in infants with P50-61; and hemorrhagic and hematological disorders specific to fetuses or newborns. In order to exclude inappropriate infants with multiple abnormal results, a multivariate iterative method called latent abnormal values exclusion (LAVE) [14] [15] [16] was applied for simultaneous derivation of RIs for multiple test items. In this study, the LAVE method was used for the values of WBC, HGB, HCT, TP, BUN, CK, K, LDH, ALT, and CRP with eight iterations and an allowance of up to one result outside the RI and up to one missing result in the reference test items.

The parametric method was used for computing the RIs after transforming the distribution of the reference values into a Gaussian form using a modified Box-Cox transformation [14]. The 90% confidence intervals (CIs) for the upper (UL) and lower limits (LL) of the RIs were estimated by the bootstrap method to avoid any abnormal results in the reference test [17, 18]. The need to partition the reference values by sex, GA, and BW was judged based on the SD ratio (SDR) introduced by Ichihara [14]; the SDR for sex (SDR_SEX_) is expressed as the SD representing the sex difference divided by the SD of the RI (SD_RI_, or 1/4th of RI). Similarly, the SDRs for GA and BW (SDR_GA_, SDR_BW_) were computed as a ratio of the SD representing the between-GA and between-BW subgroup differences divided by the SD_RI_, respectively. We adopted an SDR cutoff value of 0.4 by consensus among the collaborators [17, 19, 20].

## Results

### Outline of the Prospective Study

**Fig 1:**
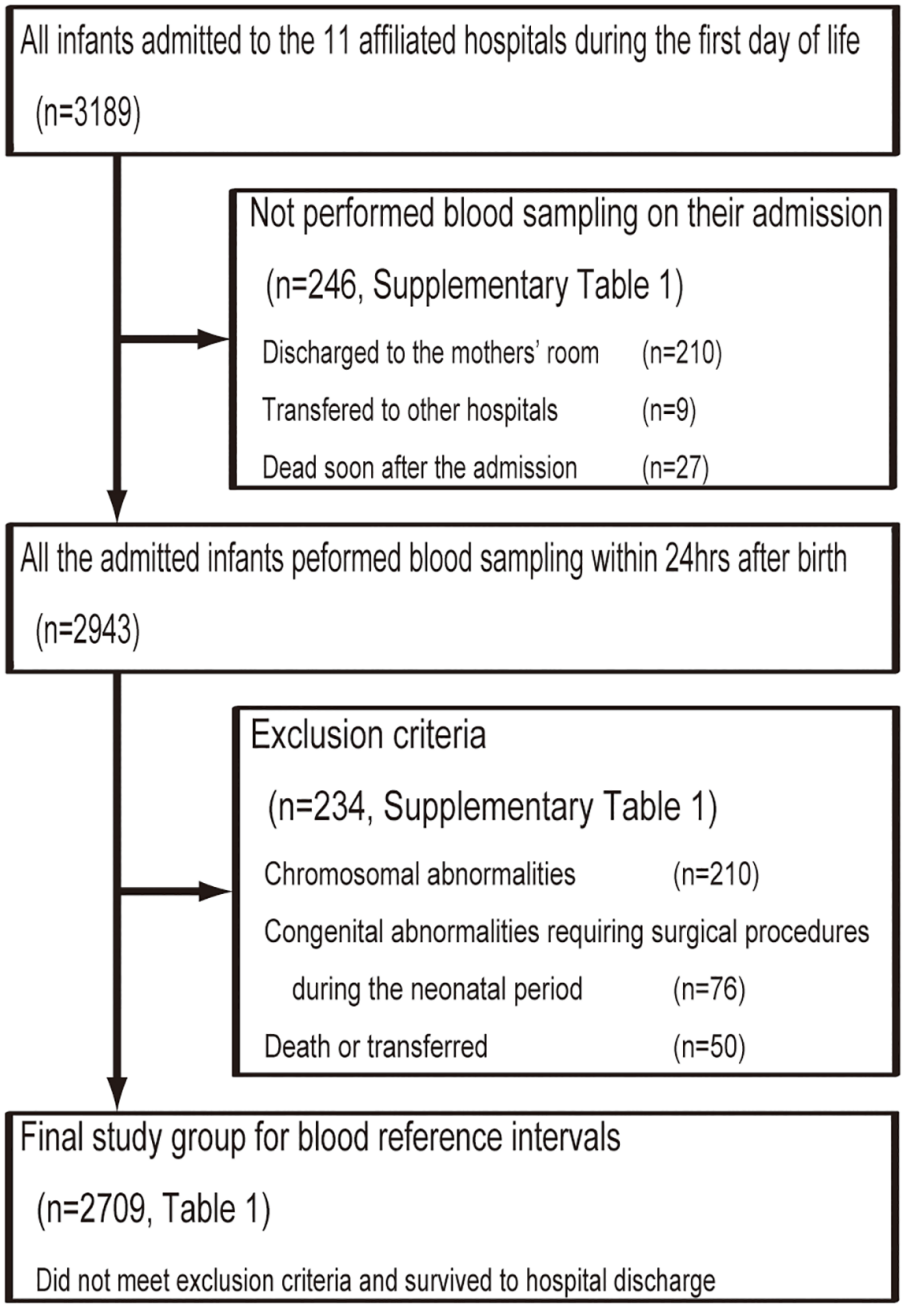
Overview of the prospective study of blood reference intervals for preterm low-birth-weight infants.

Fig 1 shows an outline for the recruitment of preterm low-birth-weight infants in this prospective study. A total of 3,189 infants were hospitalized on the first day of life at the 11 NICUs between April 2010 and March 2013; 246 did not undergo laboratory testing on admission, 210 were recognized to be healthy and did not require blood testing, 9 were transferred to other hospitals, and 27 died within 24 hours after birth. We also excluded 234 infants who received a diagnosis of congenital abnormalities (n = 210) or congenital abnormalities requiring surgical procedures within 28 days after birth (n = 76) or who died in the hospital or were transferred to other facilities (n = 50). Therefore, the final study group for analyzing the blood RIs comprised 2,709 infants who did not meet the exclusion criteria and survived until hospital discharge.

### ICD-10

Table 1 shows the number of subjects in the final group classified based on the ICD-10 code. We diagnosed 1,555 low-birth-weight infants (91.6%) (Total <2,500 g) and 1,340 preterm birth infants (89.9%) (total <37 w) with P05-08: disorders related to the length of gestation and fetal growth. S1 Table displays the ICD-10 diagnostic information for the excluded infants.

**Table 1:** Number of preterm low-birth-weight infants classified in the ICD-10 (n = 2709).

ICD-10 codes	Total <2500g				Total <37w			
		Classified by BW				Classified by GA		
		<1000g	1000-1500g	1500-2500g		<28w	28-32w	32-37w
P00-P04 Fetus and newborn affected by maternal factors and by complications of pregnancy, labour and delivery	5	0	0	5	9	0	0	9
P05-P08 Disorders related to length of gestation and fetal growth	1555	148	225	1182	1340	124	215	1001
P10-P15 Birth trauma	0	0	0	0	0	0	0	0
P20-P29 Respiratory and cardiovascular disorders specific to the perinatal period	74	0	2	72	88	0	1	87
P35-P39 Infections specific to the perinatal period	8	0	0	8	6	0	0	6
P50-P61 Haemorrhagic and haematological disorders of fetus and newborn	14	1	0	13	6	1	0	5
P70-P74 Transitory endocrine and metabolic disorders specific to fetus and newborn	34	0	3	31	31	0	1	30
P75-P78 Digestive system disorders of fetus and newborn	3	0	0	3	5	0	0	5
P80-P83 Conditions involving the integument and temperature regulation of fetus and newborn	0	0	0	0	0	0	0	0
P90-P96 Other disorders originating in the perinatal period	1	1	0	0	1	1	0	0
Q00-Q07 Congenital malformations of the nervous system	0	0	0	0	0	0	0	0
Q10-Q18 Congenital malformations of eye, ear, face and neck	1	0	0	1	1	0	0	1
Q20-Q28 Congenital malformations of the circulatory system	0	0	0	0	0	0	0	0
Q30-Q34 Congenital malformations of the respiratory system	0	0	0	0	0	0	0	0
Q35-Q37 Cleft lip and cleft palate	1	0	0	1	0	0	0	0
Q38-Q45 Other congenital malformations of the digestive system	1	0	0	1	2	0	0	2
Q50-Q56 Congenital malformations of genital organs	0	0	0	0	0	0	0	0
Q60-Q64 Congenital malformations of the urinary system	0	0	0	0	0	0	0	0
Q65-Q79 Congenital malformations and deformations of the musculoskeletal system	0	0	0	0	0	0	0	0
Q80-Q89 Other congenital malformations	0	0	0	0	0	0	0	0
Q90-Q99 Chromosomal abnormalities, not elsewhere classified	0	0	0	0	0	0	0	0
**Total**	**1697**	**150**	**230**	**1317**	**1489**	**126**	**217**	**1146**

BW; birth weight, GA; gestational age, According with the World Health Organization classification of BW and GA, the data were collected for sub-stratified by six BW and GA groups: low-birth-weight infant as a BW of <2500g, very low-birth-weight infant as <1500g, extremely low-birth-weight infant as <1000g, and moderate to late preterm infant as GA of <37w, very preterm infant as <32w, extremely preterm infant as <28w, respectively.

### GA- and BW-specific Blood RIs

**Fig 2:**
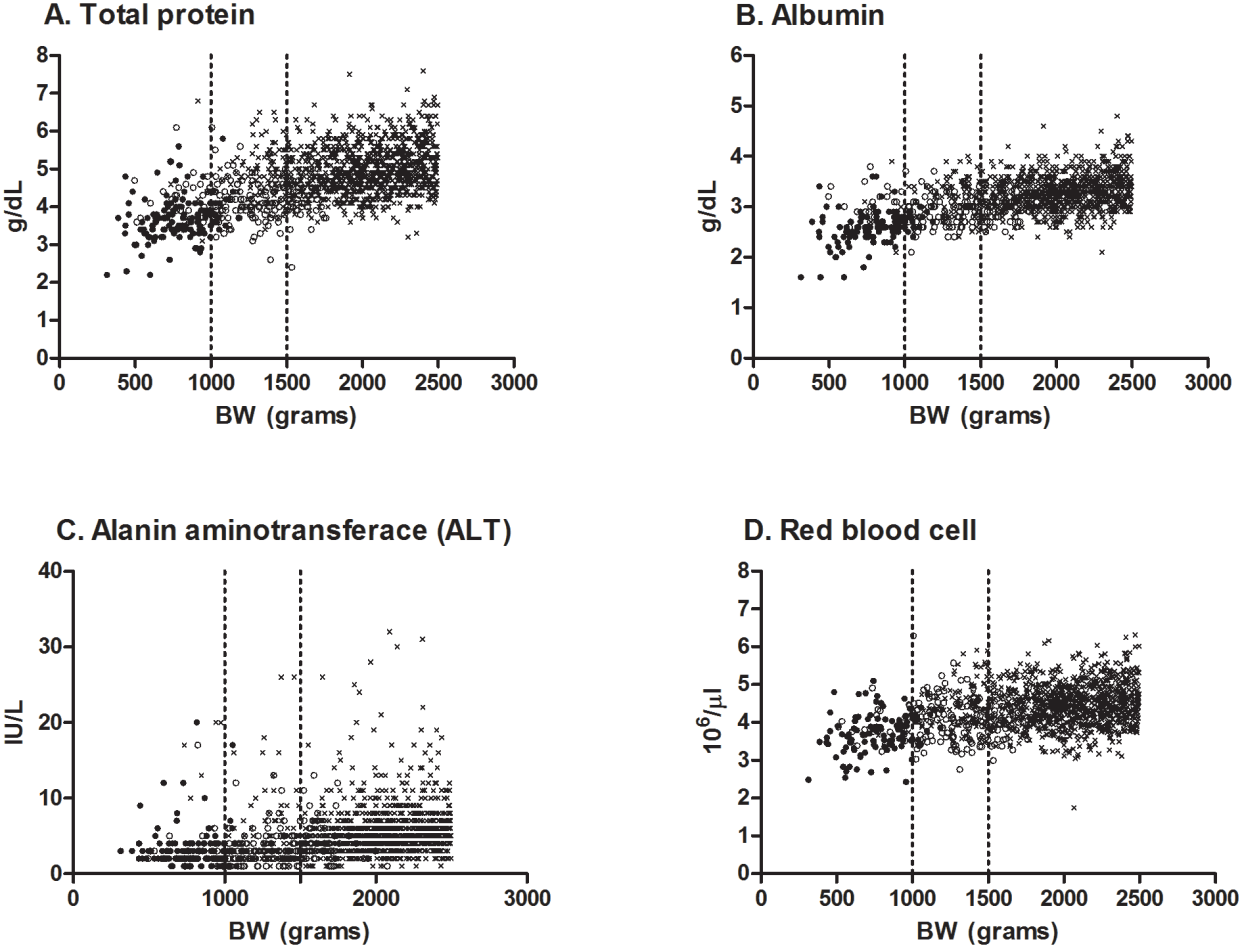
Scatter plots of the items that were significant in both the SDR_GA_ and SDR_BW_. The horizontal axis shows the birth weight (BW). The vertical axis shows the measured values of A. Total protein (g/dL), B. Albumin (g/dL), C. Alanine aminotransferace (ALT) (IU/L), and D. Red blood cells (10^6^/μl). The data were classified into three subgroups based on gestational age (GA): GA of 32–37 weeks, cross marks (×); 28–32 weeks, open circles (○); 22–28 weeks, filled circles (●).

Table 2 displays the GA-specific RIs of hematology and blood chemistry (international units). The items with SDRs of more than 0.4 were subgrouped based on the GA classification. The SDR_GA_ values of TP, ALB, CRE, Na, ALT, WBC, RBC, NEUT and MONO were significant. The BW-specific RIs for low-birth-weight infants (international units) are shown in Table 3. The SDR_BW_ values of TP, ALB, ALT, RBCs, and EOS were significant. The values of TP, ALB, ALT and RBC were significant in both SDR_GA_ and SDR_BW_, with values of more than 0.4. These levels increased in proportion to the BW and GA (Fig 2). S2 and S3 Tables show the RIs converted from international to conventional units.

**Table 2:** GA-specific RIs of blood chemistry and hematology for preterm infants (International Unit).

			Total <37w							Classified by GA																				
										<28w							28-32w							32-37w						
				LL90%CI		**RI**		UL90%CI			LL90%CI		**RI**		UL90%CI			LL90%CI		**RI**		UL90%CI			LL90%CI		**RI**		UL90%CI	
**Analyte**	**SI unit**	**SDR_GA_**	**n**	LL	UL	**LL**	**UL**	LL	UL	n	LL	UL	**LL**	**UL**	LL	UL	n	LL	UL	**LL**	**UL**	LL	UL	n	LL	UL	**LL**	**UL**	LL	UL
**TP**	**g/L**	***1*.*02***								113	25	30	**27**	**48**	44	52	194	33	35	**34**	**52**	51	54	1164	40	41	**40**	**63**	62	64
**ALB**	**g/L**	***0*.*95***								96	19	22	**20**	**32**	30	34	161	25	26	**25**	**35**	34	36	1052	28	28	**28**	**40**	39	40
**BUN**	**mmol/L**	0.14	1476	1.38	1.57	**1.44**	**5.98**	5.65	6.41																					
**CRE**	**μmol/L**	***0*.*44***								112	24.8	29.2	**27.4**	**79.7**	72.6	86.7	192	24.8	31.0	**30.1**	**79.7**	69.9	85.0	1149	35.4	37.2	**36.3**	**85.0**	81.4	87.6
**T-BIL**	**μmol/L**	0.36	1432	20.5	21.9	**21.0**	**57.8**	55.6	61.2																					
**D-BIL**	**μmol/L**	0.00	868	5.6	5.8	**5.8**	**19.2**	18.3	20.2																					
**Na**	**mmol/L**	***0*.*40***								94	128	130	**129**	**142**	141	144	162	131	133	**131**	**142**	141	143	1050	133	134	**134**	**142**	142	143
**K**	**mmol/L**	0.00	1272	3.6	3.7	**3.7**	**6.1**	6.0	6.2																					
**CL**	**mmol/L**	0.00	1314	100	101	**101**	**111**	111	112																					
**Ca**	**mmol/L**	0.18	1466	2.0	2.0	**2.0**	**2.7**	2.6	2.7																					
**CRP**	**μg/L**	0.14	1356	0.00	0.00	**0.00**	**0.80**	0.20	7.20																					
**AST**	**IU/L**	0.28	1281	15	16	**16**	**70**	65	78																					
**ALT**	**IU/L**	***0*.*66***								105	1	1	**1**	**6**	5	9	193	1	1	**1**	**9**	7	11	1149	2	2	**2**	**12**	11	13
**LDH**	**IU/L**	0.11	1297	238	263	**245**	**787**	742	958																					
**ALP**	**IU/L**	0.16	462	351	397	**372**	**1082**	1022	1152																					
**CK**	**IU/L**	***0*.*44***								88	22	60	**45**	**916**	583	1263	173	38	76	**53**	**552**	455	694	988	79	113	**91**	**707**	644	778
**WBC**	**10**^**9**^**/L**	***0*.*57***								102	2.55	3.87	**3.04**	**31.0**	22.5	39.2	179	2.58	4.07	**3.40**	**19.9**	16.4	23.5	1100	5.76	6.75	**6.03**	**21.2**	20.3	22.6
**RBC**	**10**^**12**^**/L**	***0*.*59***								103	2.63	2.98	**2.76**	**4.65**	4.51	4.82	178	3.20	3.41	**3.28**	**5.07**	4.91	5.27	1084	3.59	3.65	**3.62**	**5.52**	5.46	5.57
**HGB**	**g/L**	0.33	1406	125	129	**127**	**203**	201	206																					
**HCT**	**/L**	0.31	1408	0.37	0.38	**0.38**	**0.60**	0.60	0.61																					
**PLT**	**10**^**9**^**/L**	0.20	1344	107	120	**114**	**375**	367	382																					
**NEUT**	**10**^**9**^**/L**	***0*.*79***								46	0.17	0.81	**0.64**	**17.0**	11.7	27.6	91	0.15	0.68	**0.37**	**9.58**	7.36	12.6	490	0.93	1.54	**1.19**	**12.7**	10.7	14.3
**LYMP**	**10**^**9**^**/L**	0.30	754	1.88	2.36	**2.06**	**9.72**	9.21	10.3																					
**MONO**	**10**^**6**^**/L**	***0*.*64***								52	30	152	**57**	**1623**	1270	2185	118	29	110	**64**	**1147**	1005	1323	578	150	217	**181**	**1413**	1286	1523
**EOS**	**10**^**6**^**/L**	0.32	745	6	36	**8**	**741**	693	797																					
**BASO**	**10**^**6**^**/L**	0.10	748	1	2	**2**	**270**	241	300																					

TP; total protein, ALB; albumin, BUN; blood urea nitrogen, CRE; creatinine, T-BIL; total bilirubin, D-BIL; direct bilirubin, Na; sodium, K; potassium, CL; chlorine, Ca; calcium, CRP; C-reactive protein, AST; aspartate aminotransferase, ALT; alanine aminotransferase, LDH; lactate dehydrogenase, ALP; alkaline phosphatase, CK; creatine kinase, WBC; white blood cell, RBC; red blood cell, HGB; hemoglobin, HCT; hematocrit, PLT; platelet, NEUT; neutrophil, LYMP; lymphocyte, MONO; monocyte, EOS; eosinophil, BASO; basophil, GA; gestational age, RI; reference interval, LL; lower limit of the RI, UL; upper limit of the RI, CI; confidential interval (90%) of LLs and ULs were estimated by the bootstrap method. The results were excluded data of LDH, AST and CK in the infants with P20-29; Respiratory and cardiovascular disorders specific to the perinatal period, CRP with P35-39; Infections specific to the perinatal period, and K and T-Bil with P50-61; Hemorrhagic and homological disorders specific to fetus and newborn, respectively. A multivariate iterative method called latent abnormal value exclusion (LAVE) was applied to nine analytes (TP, BUN, K, LDH, ALT, WBC, CRP, HGB and HCT) which deemed to be adversely affected by hemolysis and inflammation. By use of 3-level nested ANOVA, the influence of GA on test results was expressed in terms of standard deviation (SD) ratio (SDR), as SDR_GA_. SDR> = 0.4 were used as a criteria for the need of partition by the factor.

**Table 3:** BW-specific RIs of blood chemistry and hematology for low-birth-weight infants (International Unit)

			Total <2500g							Classified by BW																				
										<1000g							1000-1500g							1500-2500g						
				LL90%CI		**RI**		UL90%CI			LL90%CI		**RI**		UL90%CI			LL90%CI		**RI**		UL90%CI			LL90%CI		**RI**		UL90%CI	
**Analyte**	**SI unit**	**SDR_BW_**	n	LL	UL	**LL**	**UL**	LL	UL	n	LL	UL	**LL**	**UL**	LL	UL	n	LL	UL	**LL**	**UL**	LL	UL	n	LL	UL	**LL**	**UL**	LL	UL
**TP**	**g/L**	***0*.*94***								132	26	31	**29**	**51**	48	57	202	33	35	**34**	**60**	58	63	1178	40	41	**40**	**64**	63	65
**ALB**	**g/L**	***0*.*89***								112	19	22	**21**	**35**	33	37	169	25	25	**25**	**38**	37	39	1010	28	28	**28**	**40**	40	41
**BUN**	**mmol/L**	0.00	1532	1.38	1.59	**1.46**	**6.12**	5.78	6.51																					
**CRE**	**μmol/L**	0.32	1528	31.0	35.4	**31.9**	**85.0**	81.4	92.0																					
**T-BIL**	**μmol/L**	0.11	1474	20.5	22.1	**21.2**	**60.7**	58.0	64.6																					
**D-BIL**	**μmol/L**	0.00	917	5.5	5.8	**5.6**	**20.0**	19.0	21.0																					
**Na**	**mmol/L**	0.38	1315	132	133	**133**	**143**	142	143																					
**K**	**mmol/L**	0.00	1287	3.6	3.7	**3.7**	**6.1**	6.0	6.2																					
**CL**	**mmol/L**	0.13	1315	100	101	**101**	**111**	111	112																					
**Ca**	**mmol/L**	0.10	1519	2.0	2.0	**2.0**	**2.7**	2.6	2.7																					
**CRP**	**μg/L**	0.00	1521	0.0	0.0	**0.0**	**5.1**	0.3	7.7																					
**AST**	**IU/L**	0.00	1268	15	16	**15**	**74**	70	82																					
**ALT**	**IU/L**	***0*.*49***								120	1	1	**1**	**7**	6	12	202	1	1	**1**	**13**	9	17	1161	2	2	**2**	**12**	11	13
**LDH**	**IU/L**	0.00	1281	235	261	**243**	**835**	767	984																					
**ALP**	**IU/L**	0.00	463	352	393	**372**	**1097**	1051	1181																					
**CK**	**IU/L**	0.34	1258	58	73	**64**	**685**	641	744																					
**WBC**	**10**^**9**^**/L**	0.22	1437	4.13	4.91	**4.42**	**24.0**	23.0	25.6																					
**RBC**	**10**^**12**^**/L**	***0*.*59***								115	2.68	3.00	**2.86**	**4.61**	4.48	4.79	189	3.23	3.47	**3.29**	**5.38**	5.22	5.65	1088	3.62	3.70	**3.65**	**5.58**	5.52	5.66
**HGB**	**g/L**	0.36	1450	125	129	**127**	**204**	203	206																					
**HCT**	**/L**	0.29	1454	0.37	0.39	**0.38**	**0.61**	0.60	0.61																					
**PLT**	**10**^**9**^**/L**	0.21	1392	108	121	**108**	**375**	365	383																					
**NEUT**	**10**^**9**^**/L**	0.27	748	0.74	1.07	**0.90**	**17.5**	15.9	19.1																					
**LYMP**	**10**^**9**^**/L**	0.00	877	1.67	2.15	**1.84**	**9.37**	8.93	9.78																					
**MONO**	**10**^**6**^**/L**	0.21	875	116	164	**143**	**1599**																							
**EOS**	**10**^**6**^**/L**	***0*.*42***								62	1	24	**5**	**411**	289	552	112	0	6	**0**	**555**	462	755	596	45	73	**59**	**867**	778	932
**BASO**	**10**^**6**^**/L**	0.09	823	1	3	**2**	**284**	249	313																					

TP; total protein, ALB; albumin, BUN; blood urea nitrogen, CRE; creatinine, T-BIL; total bilirubin, D-BIL; direct bilirubin, Na; sodium, K; potassium, CL; chlorine, Ca; calcium, CRP; C-reactive protein, AST; aspartate aminotransferase, ALT; alanine aminotransferase, LDH; lactate dehydrogenase, ALP; alkaline phosphatase, CK; creatine kinase, WBC; white blood cell, RBC; red blood cell, HGB; hemoglobin, HCT; hematocrit, PLT; platelet, NEUT; neutrophil, LYMP; lymphocyte, MONO; monocyte, EOS; eosinophil, BASO; basophil, GA; gestational age, RI; reference interval, LL; lower limit of the RI, UL; upper limit of the RI, CI; confidential interval (90%) of LLs and ULs were estimated by the bootstrap method. The results were excluded data of LDH, AST and CK in the infants with P20-29; Respiratory and cardiovascular disorders specific to the perinatal period, CRP with P35-39; Infections specific to the perinatal period, and K and T-Bil with P50-61; Hemorrhagic and homological disorders specific to fetus and newborn, respectively. A multivariate iterative method called latent abnormal value exclusion (LAVE) was applied to nine analytes (TP, BUN, K, LDH, ALT, WBC, CRP, HGB and HCT) which deemed to be adversely affected by hemolysis and inflammation. By use of 3-level nested ANOVA, the influence of BW on test results was expressed in terms of standard deviation (SD) ratio (SDR), as SDR_BW_. SDR> = 0.4 were used as a criteria for the need of partition by the factor.

## Discussion

The Kyushu University High-Risk Neonatal Clinical Research Network Project recently established a university initiative to accumulate information for all infants admitted to the affiliated NICUs using a web-based electronic medical software program. As part of this initiative, our study’s main purpose was to establish RIs for hematology and blood chemistry analytes in preterm low-birth-weight infants who were admitted to perinatal-neonatal care centers in Japan and who survived until discharge.

We were able to obtain a sufficient sample size (2709 infants) during the three-year study period. The practically attainable target sample size for each analyte was set at a minimum of 250 or more, which is greater than twice the minimum number (120 or more) [19]. We excluded outliers based on the clinical diagnosis (ICD-10) and the results of the multivariate iterative method (LAVE) [19]. The LAVE features the simultaneous setting of RIs for multiple test items that are mutually related and the rigid exclusion of individuals with abnormal values for other test items [20]. As a result, the reference individuals were “considered to be normal” preterm low-birth-weight infants based on the ICD-10 code (Table 1).

The Ichihara method utilizes information for the SDR attributable to each source of variation and can be applied in situations in which more than two subgroups are categorized according to the factors [15] [18]. In our study, we presented each RI classified into six subgroups by the BW and GA according to the SDR_BW_ and SDR_GA_, respectively. The SDR_SEX_ of each item were zero for all analytes for the age groups (data not shown). Therefore, sex-specific RIs were not presented in this study.

In our study, the following tended to increase in proportion to both the GA and BW: TP, Alb, and ALT (Tables 2 and 3 and Fig 2). The TP is made up of Alb and globulin. Alb is synthesized in the liver, and a low serum Alb may result from immaturity of the liver function. ALT is an important transaminase enzyme in various tissue, especially the liver; therefore, the blood ALT level is used clinically as a biomarker for the liver function. In small-for-gestational-age infants, the AST and ALT serum activities were correlated with BW and GA [21]. Our data confirmed the parallel upward trend in these values as the organ function matured. In contrast, extremely premature infants had high plasma enzyme activities compared to babies at a later corrected GA [22], possibly due to suffering more severe illness immediately after birth.

Significant SDR_GA_ and SDR_BW_ values (>0.4) were observed for WBC, RBC, NEUT, and MONO (Table 2); and RBC and EO (Table 3), respectively. When the test results of those analytes were compared among the groups, the RBC count was found to have a tendency to increase in proportion to both the BW and GA (Fig 2). The RIs of HCT and HGB in extremely preterm patients were reported to be lower than those in later preterm and term infants [23]. Several reports have shown the same gradual upward tendency in hematological data [9] [23]. The PLT counts increased for GAs of 22 to 42 weeks using a huge data system [8]. In contrast, an abnormal lymphocyte count at birth is associated with adverse outcomes, including early-onset sepsis, intraventricular hemorrhaging and retinopathy of prematurity [24]. The onset of neutropenia in the first days of life is sometimes noted in SGA infants or those born to mothers with persistent maternal hypertension or early-onset bacterial infection [25]. These reports suggest that the GA and BW, as well as potentially pathogenic maternal and neonatal variables, should be considered when developing RIs.

We recognize various limitations and pitfalls that should be considered when applying these RIs in practice. First, preterm or low-birth-weight babies are considered to be in a clinically pathological or unhealthy state, and many require medical management. Therefore, “normal range” is not a suitable term for the blood chemistry and hematology data for these infants. We therefore used the term “reference interval” in this project and discarded data confirmed to be unacceptable based on the ICD-10 code and LAVE method. A second limitation is that the source of blood specimens (capillary, venous, or arterial) was not taken into account. Some hematological and chemical test values are somewhat higher in capillary samples than in venous or arterial samples. The third limitation is that we did not analyze the trends in the values after birth [23]. The values of analytes may change after several postnatal days depending on the clinical course of the infant.

## Conclusions

Our project provides 26 blood RIs in preterm low-birth-weight infants requiring neonatal intensive care in Japan. These RIs should help researchers in the field of perinatal-neonatal medicine perform proper assessments in routine clinical work and research. Further evaluations are needed to determine whether these RIs are representative of the physiological data for those infants.

## Supporting Information

S1 Table The ICD-10 of the excluded infants (DOCX)

S2 Table GA-specific RIs of blood chemistry and hematology for preterm infants (Conventional Unit) (DOCX)

S3 Table BW-specific RIs of blood chemistry and hematology for low birth weight infants (Conventional Unit) (DOCX)

## References

[1] Itabashi K, Horiuchi T, Kusuda S, Kabe K, Itani Y, Nakamura T, et al Mortality rates for extremely low birth weight infants born in Japan in 2005. Pediatrics. 2009;123(2):445–50. Epub 2009/01/28. 123/2/445 [pii] 10.1542/peds.2008-0763 . 19171608

[2] Ishii N, Kono Y, Yonemoto N, Kusuda S, Fujimura M. Outcomes of infants born at 22 and 23 weeks' gestation. Pediatrics. 2013;132(1):62–71. Epub 2013/06/05. 10.1542/peds.2012-2857 peds.2012-2857 [pii]. . 23733804

[3] Fleming S, Thompson M, Stevens R, Heneghan C, Pluddemann A, Maconochie I, et al Normal ranges of heart rate and respiratory rate in children from birth to 18 years of age: a systematic review of observational studies. Lancet. 2011;377(9770):1011–8. Epub 2011/03/18. 10.1016/S0140-6736(10)62226-X S0140-6736(10)62226-X [pii]. 21411136 PMC3789232

[4] Jung B, Adeli K. Clinical laboratory reference intervals in pediatrics: the CALIPER initiative. Clin Biochem. 2009;42(16–17):1589–95. Epub 2009/07/14. 10.1016/j.clinbiochem.2009.06.025 S0009-9120(09)00293-8 [pii]. . 19591815

[5] Colantonio DA, Kyriakopoulou L, Chan MK, Daly CH, Brinc D, Venner AA, et al Closing the gaps in pediatric laboratory reference intervals: a CALIPER database of 40 biochemical markers in a healthy and multiethnic population of children. Clin Chem. 2012;58(5):854–68. Epub 2012/03/01. 10.1373/clinchem.2011.177741 clinchem.2011.177741 [pii]. . 22371482

[6] Schmutz N, Henry E, Jopling J, Christensen RD. Expected ranges for blood neutrophil concentrations of neonates: the Manroe and Mouzinho charts revisited. J Perinatol. 2008;28(4):275–81. Epub 2008/01/18. 10.1038/sj.jp.7211916 7211916 [pii]. . 18200025

[7] Christensen RD, Jopling J, Henry E, Wiedmeier SE. The erythrocyte indices of neonates, defined using data from over 12,000 patients in a multihospital health care system. J Perinatol. 2008;28(1):24–8. Epub 2007/11/02. 7211852 [pii] 10.1038/sj.jp.7211852 . 17972890

[8] Wiedmeier SE, Henry E, Sola-Visner MC, Christensen RD. Platelet reference ranges for neonates, defined using data from over 47,000 patients in a multihospital healthcare system. J Perinatol. 2009;29(2):130–6. Epub 2008/09/27. 10.1038/jp.2008.141 jp2008141 [pii]. . 18818663

[9] Jopling J, Henry E, Wiedmeier SE, Christensen RD. Reference ranges for hematocrit and blood hemoglobin concentration during the neonatal period: data from a multihospital health care system. Pediatrics. 2009;123(2):e333–7. Epub 2009/01/28. 10.1542/peds.2008-2654 peds.2008-2654 [pii]. . 19171584

[10] Chan MK, Seiden-Long I, Aytekin M, Quinn F, Ravalico T, Ambruster D, et al Canadian Laboratory Initiative on Pediatric Reference Interval Database (CALIPER): pediatric reference intervals for an integrated clinical chemistry and immunoassay analyzer, Abbott ARCHITECT ci8200. Clin Biochem. 2009;42(9):885–91. Epub 2009/03/26. 10.1016/j.clinbiochem.2009.01.014 S0009-9120(09)00046-0 [pii]. . 19318027

[11] Demirel G, Celik IH, Canpolat FE, Erdeve O, Biyikli Z, Dilmen U. Reference values of serum cystatin C in very low-birthweight premature infants. Acta Paediatr. 2013;102(1):e4–7. Epub 2012/09/29. 10.1111/apa.12041 . 23016830

[12] Vieux R, Hascoet JM, Merdariu D, Fresson J, Guillemin F. Glomerular filtration rate reference values in very preterm infants. Pediatrics. 2010;125(5):e1186–92. Epub 2010/04/07. 10.1542/peds.2009-1426 peds.2009-1426 [pii]. . 20368313

[13] Fenton TR, Lyon AW, Rose MS. Cord blood calcium, phosphate, magnesium, and alkaline phosphatase gestational age-specific reference intervals for preterm infants. BMC Pediatr. 2011;11:76 Epub 2011/09/03. 10.1186/1471-2431-11-76 1471-2431-11-76 [pii]. 21884590 PMC3179922

[14] Ichihara K, Yomamoto Y, Hotta T, Hosogaya S, Miyachi H, Itoh Y, et al Collaborative derivation of reference intervals for major clinical laboratory tests in Japan. Ann Clin Biochem. 2015 Epub 2015/09/13. 0004563215608875 [pii] 10.1177/0004563215608875 . 26362325

[15] Kinoshita S, Toyofuku M, Iida H, Wakiyama M, Kurihara M, Nakahara M, et al Standardization of laboratory data and establishment of reference intervals in the Fukuoka Prefecture: a Japanese perspective. Clin Chem Lab Med. 2001;39(3):256–62. Epub 2001/05/15. 10.1515/CCLM.2001.040 . 11350024

[16] Ichihara K, Ozarda Y, Klee G, Straseski J, Baumann N, Ishikura K. Utility of a panel of sera for the alignment of test results in the worldwide multicenter study on reference values. Clin Chem Lab Med. 2013;51(5):1007–25. Epub 2013/05/02. 10.1515/cclm-2013-0248 /j/cclm.2013.51.issue-5/cclm-2013-0248/cclm-2013-0248.xml [pii] /j/cclm.ahead-of-print/cclm-2013-0248/cclm-2013-0248.xml [pii]. . 23633468

[17] Yamakado M, Ichihara K, Matsumoto Y, Ishikawa Y, Kato K, Komatsubara Y, et al Derivation of gender and age-specific reference intervals from fully normal Japanese individuals and the implications for health screening. Clin Chim Acta. 2015;447:105–14. Epub 2015/05/20. 10.1016/j.cca.2015.04.037 S0009-8981(15)00236-3 [pii]. . 25987309

[18] Ozarda Y, Ichihara K, Aslan D, Aybek H, Ari Z, Taneli F, et al A multicenter nationwide reference intervals study for common biochemical analytes in Turkey using Abbott analyzers. Clin Chem Lab Med. 2014;52(12):1823–33. Epub 2014/08/26. 10.1515/cclm-2014-0228 /j/cclm.2014.52.issue-12/cclm-2014-0228/cclm-2014-0228.xml [pii] /j/cclm.ahead-of-print/cclm-2014-0228/cclm-2014-0228.xml [pii]. . 25153598

[19] Ichihara K, Boyd JC. An appraisal of statistical procedures used in derivation of reference intervals. Clin Chem Lab Med. 2010;48(11):1537–51. Epub 2010/11/11. 10.1515/CCLM.2010.319 . 21062226

[20] Ichihara K. Statistical considerations for harmonization of the global multicenter study on reference values. Clin Chim Acta. 2014;432:108–18. Epub 2014/02/13. 10.1016/j.cca.2014.01.025 S0009-8981(14)00042-4 [pii]. . 24518360

[21] Zanardo V, Perini G. Aspartate aminotransferase and alanine aminotransferase serum activities in small-for-date newborns. Padiatr Padol. 1987;22(4):325–30. Epub 1987/01/01. . 3438090

[22] Victor S, Dickinson H, Turner MA. Plasma aminotransferase concentrations in preterm infants. Arch Dis Child Fetal Neonatal Ed. 2011;96(2):F144–5. Epub 2009/07/04. 10.1136/adc.2008.152454 adc.2008.152454 [pii]. . 19574257

[23] Christensen RD, Henry E, Jopling J, Wiedmeier SE. The CBC: reference ranges for neonates. Semin Perinatol. 2009;33(1):3–11. Epub 2009/01/27. 10.1053/j.semperi.2008.10.010 S0146-0005(08)00127-4 [pii]. . 19167576

[24] Christensen RD, Baer VL, Gordon PV, Henry E, Whitaker C, Andres RL, et al Reference ranges for lymphocyte counts of neonates: associations between abnormal counts and outcomes. Pediatrics. 2012;129(5):e1165–72. Epub 2012/04/18. 10.1542/peds.2011-2661 peds.2011-2661 [pii]. . 22508916

[25] Christensen RD, Henry E, Wiedmeier SE, Stoddard RA, Lambert DK. Low blood neutrophil concentrations among extremely low birth weight neonates: data from a multihospital health-care system. J Perinatol. 2006;26(11):682–7. Epub 2006/10/13. 7211603 [pii] 10.1038/sj.jp.7211603 . 17036034

