# Supplementary material for: Blood Reference Intervals for Preterm Low-Birth-Weight Infants: A Multicenter Cohort Study in Japan
Source: PLoS One. 2016 Aug 23;11(8):e0161439. doi: 10.1371/journal.pone.0161439 (PMC4994999; doi:10.1371/journal.pone.0161439)
Supplement: S1 Table — (DOCX) [file pone.0161439.s001.docx]

**Supporting Information**

**S1 Table. The ICD-10 of the excluded infants**

| **ICD-10 codes** | *Not examined (n=246) | | #Exclusion criteria (n=234) | | |  |
| --- | --- | --- | --- | --- | --- | --- |
|  | n | % | | n | % | |
| P00-P04　Fetus and newborn affected by maternal factors and by complications of pregnancy, labour and delivery | 10 | 4.1 | | 0 | 0.0 | |
| P05-P08　Disorders related to length of gestation and fetal growth | 132 | 53.7 | | 22 | 9.4 | |
| P10-P15　Birth trauma | 2 | 0.8 | | 0 | 0.0 | |
| P20-P29　Respiratory and cardiovascular disorders specific to the perinatal period | 21 | 8.5 | | 2 | 0.9 | |
| P35-P39　Infections specific to the perinatal period | 8 | 3.3 | | 2 | 0.9 | |
| P50-P61　Haemorrhagic and haematological disorders of fetus and newborn | 12 | 4.9 | | 1 | 0.4 | |
| P70-P74　Transitory endocrine and metabolic disorders specific to fetus and newborn | 1 | 0.4 | | 0 | 0.0 | |
| P75-P78　Digestive system disorders of fetus and newborn | 8 | 3.3 | | 0 | 0.0 | |
| P80-P83　Conditions involving the integument and temperature regulation of fetus and newborn | 2 | 0.8 | | 8 | 3.4 | |
| P90-P96　Other disorders originating in the perinatal period | 0 | 0.0 | | 0 | 0.0 | |
| Q00-Q07　Congenital malformations of the nervous system | 4 | 1.6 | | 23 | 9.8 | |
| Q10-Q18　Congenital malformations of eye, ear, face and neck | 0 | 0.0 | | 1 | 0.4 | |
| Q20-Q28　Congenital malformations of the circulatory system | 25 | 10.2 | | 82 | 35.0 | |
| Q30-Q34　Congenital malformations of the respiratory system | 2 | 0.8 | | 6 | 2.6 | |
| Q35-Q37　Cleft lip and cleft palate | 2 | 0.8 | | 0 | 0.0 | |
| Q38-Q45　Other congenital malformations of the digestive system | 4 | 1.6 | | 33 | 14.1 | |
| Q50-Q56　Congenital malformations of genital organs | 0 | 0.0 | | 1 | 0.4 | |
| Q60-Q64　Congenital malformations of the urinary system | 4 | 1.6 | | 0 | 0.0 | |
| Q65-Q79　Congenital malformations and deformations of the musculoskeletal system | 1 | 0.4 | | 7 | 3.0 | |
| Q80-Q89　Other congenital malformations | 4 | 1.6 | | 6 | 2.6 | |
| Q90-Q99　Chromosomal abnormalities, not elsewhere classified | 4 | 1.9 | | 40 | 17.1 | |

*We did not perform blood sampling in 246 infants on their admission; 210 of them were discharge to the mother’s room, and 9 and 27 were transferred to other hospital and dead soon after admission. #We excluded 234 infants who had a diagnosis of congenital abnormalities (N=210), congenital abnormalities requiring surgical procedures during the neonatal period (N=76), and died or transferred (N=50).
