# Supplementary material for: Blood Reference Intervals for Preterm Low-Birth-Weight Infants: A Multicenter Cohort Study in Japan
Source: PLoS One. 2016 Aug 23;11(8):e0161439. doi: 10.1371/journal.pone.0161439 (PMC4994999; doi:10.1371/journal.pone.0161439)
Supplement: S2 Table — (DOCX) [file pone.0161439.s002.docx]

**Supporting Information**

**S2 Table. GA-specific RIs of blood chemistry and hematology for preterm infants (Conventional Unit)**

|  | | | **Total <37w** | | | | | | | **Classified by GA** | | | | | | | | | | | | | | | | | | | | |
| --- | --- | --- | --- | --- | --- | --- | --- | --- | --- | --- | --- | --- | --- | --- | --- | --- | --- | --- | --- | --- | --- | --- | --- | --- | --- | --- | --- | --- | --- | --- |
|  |  |  |  |  |  |  |  |  |  | **<28w** | | | | | | | **28-32w** | | | | | | | **32-37w** | | | | | | |
|  |  |  |  | LL90%CI | | **RI** | | UL90%CI | |  | LL90%CI | | **RI** | | UL90%CI | |  | LL90%CI | | **RI** | | UL90%CI | |  | LL90%CI | | **RI** | | UL90%CI | |
| **Analyte** | **Unit** | **SDR_GA_** | n | LL | UL | **LL** | **UL** | LL | UL | n | LL | UL | **LL** | **UL** | LL | UL | n | LL | UL | **LL** | **UL** | LL | UL | n | LL | UL | **LL** | **UL** | LL | UL |
| **TP** | **g/L** | ***1.02*** |  |  |  |  |  |  |  | 113 | 2.5 | 3.0 | **2.7** | **4.8** | 4.4 | 5.2 | 194 | 3.3 | 3.5 | **3.4** | **5.2** | 5.1 | 5.4 | 1164 | 4.0 | 4.1 | **4.0** | **6.3** | 6.2 | 6.4 |
| **ALB** | **g/L** | ***0.95*** |  |  |  |  |  |  |  | 96 | 1.9 | 2.2 | **2.2** | **3.2** | 3.0 | 3.4 | 161 | 2.5 | 2.6 | **2.5** | **3.5** | 3.4 | 3.6 | 1052 | 2.8 | 2.8 | **2.8** | **4.0** | 3.9 | 4.0 |
| **BUN** | **mg/dL** | 0.14 | 1476 | 3.8 | 4.3 | **4.0** | **16.5** | 15.6 | 17.7 |  |  |  |  |  |  |  |  |  |  |  |  |  |  |  |  |  |  |  |  |  |
| **CRE** | **mg/dL** | ***0.44*** |  |  |  |  |  |  |  | 112 | 0.28 | 0.33 | **0.31** | **0.90** | 0.82 | 0.98 | 192 | 0.28 | 0.35 | **0.34** | **0.90** | 0.79 | 0.96 | 1149 | 0.40 | 0.42 | **0.41** | **0.96** | 0.92 | 0.99 |
| **T-BIL** | **mg/dL** | 0.36 | 1432 | 1.2 | 1.3 | **1.2** | **3.4** | 3.3 | 3.6 |  |  |  |  |  |  |  |  |  |  |  |  |  |  |  |  |  |  |  |  |  |
| **D-BIL** | **mg/dL** | 0.00 | 868 | 0.3 | 0.3 | **0.3** | **1.2** | 1.1 | 1.2 |  |  |  |  |  |  |  |  |  |  |  |  |  |  |  |  |  |  |  |  |  |
| **Na** | **mEq/L** | ***0.40*** |  |  |  |  |  |  |  | 94 | 128 | 130 | **129** | **142** | 141 | 144 | 162 | 131 | 133 | **131** | **142** | 141 | 143 | 1050 | 133 | 134 | **134** | **142** | 142 | 143 |
| **K** | **mEq/L** | 0.00 | 1272 | 3.6 | 3.7 | **3.7** | **6.1** | 6.0 | 6.2 |  |  |  |  |  |  |  |  |  |  |  |  |  |  |  |  |  |  |  |  |  |
| **CL** | **mEq/L** | 0.00 | 1314 | 100 | 101 | **101** | **111** | 111 | 112 |  |  |  |  |  |  |  |  |  |  |  |  |  |  |  |  |  |  |  |  |  |
| **Ca** | **mg/dL** | 0.18 | 1466 | 8.0 | 8.1 | **8.1** | **10.6** | 10.6 | 10.7 |  |  |  |  |  |  |  |  |  |  |  |  |  |  |  |  |  |  |  |  |  |
| **CRP** | **mg/dL** | 0.14 | 1356 | 0.00 | 0.00 | **0.00** | **0.08** | 0.02 | 0.72 |  |  |  |  |  |  |  |  |  |  |  |  |  |  |  |  |  |  |  |  |  |
| **AST** | **IU/L** | 0.28 | 1281 | 15 | 16 | **16** | **70** | 65 | 78 |  |  |  |  |  |  |  |  |  |  |  |  |  |  |  |  |  |  |  |  |  |
| **ALT** | **IU/L** | ***0.66*** |  |  |  |  |  |  |  | 105 | 1 | 1 | **1** | **6** | 5 | 9 | 193 | 1 | 1 | **1** | **9** | 7 | 11 | 1149 | 2 | 2 | **2** | **12** | 11 | 13 |
| **LDH** | **IU/L** | 0.11 | 1297 | 238 | 263 | **245** | **787** | 742 | 958 |  |  |  |  |  |  |  |  |  |  |  |  |  |  |  |  |  |  |  |  |  |
| **ALP** | **IU/L** | 0.16 | 462 | 351 | 397 | **372** | **1082** | 1022 | 1152 |  |  |  |  |  |  |  |  |  |  |  |  |  |  |  |  |  |  |  |  |  |
| **CK** | **IU/L** | ***0.44*** |  |  |  |  |  |  |  | 88 | 22 | 60 | **45** | **916** | 583 | 1263 | 173 | 38 | 76 | **53** | **552** | 455 | 694 | 988 | 79 | 113 | **91** | **707** | 644 | 778 |
| **WBC** | **10^3^/μL** | ***0.57*** |  |  |  |  |  |  |  | 102 | 2.55 | 3.87 | **3.04** | **31.0** | 22.5 | 39.2 | 179 | 2.58 | 4.07 | **3.40** | **19.9** | 16.4 | 23.5 | 1100 | 5.76 | 6.75 | **6.03** | **21.2** | 20.3 | 22.6 |
| **RBC** | **10^6^/μL** | ***0.59*** |  |  |  |  |  |  |  | 103 | 2.63 | 2.98 | **2.76** | **4.65** | 4.51 | 4.82 | 178 | 3.20 | 3.41 | **3.28** | **5.07** | 4.91 | 5.27 | 1084 | 3.59 | 3.65 | **3.62** | **5.52** | 5.46 | 5.57 |
| **HGB** | **g/dL** | 0.33 | 1406 | 12.5 | 12.9 | **12.7** | **20.3** | 20.1 | 20.6 |  |  |  |  |  |  |  |  |  |  |  |  |  |  |  |  |  |  |  |  |  |
| **HCT** | **%** | 0.31 | 1408 | 37.3 | 38.8 | **38.0** | **60.2** | 59.6 | 61.0 |  |  |  |  |  |  |  |  |  |  |  |  |  |  |  |  |  |  |  |  |  |
| **PLT** | **10^3^/μL** | 0.20 | 1344 | 107 | 120 | **114** | **375** | 367 | 382 |  |  |  |  |  |  |  |  |  |  |  |  |  |  |  |  |  |  |  |  |  |
| **NEUT** | **10^3^/μL** | ***0.79*** |  |  |  |  |  |  |  | 46 | 0.17 | 0.81 | **0.64** | **17.0** | 11.7 | 27.6 | 91 | 0.15 | 0.68 | **0.37** | **9.58** | 7.36 | 12.6 | 490 | 0.93 | 1.54 | **1.19** | **12.7** | 10.7 | 14.3 |
| **LYMP** | **10^3^/μL** | 0.30 | 754 | 1.88 | 2.36 | **2.06** | **9.72** | 9.21 | 10.3 |  |  |  |  |  |  |  |  |  |  |  |  |  |  |  |  |  |  |  |  |  |
| **MONO** | **/μL** | ***0.64*** |  |  |  |  |  |  |  | 52 | 30 | 152 | **57** | **1623** | 1270 | 2185 | 118 | 29 | 110 | **64** | **1147** | 1005 | 1323 | 578 | 150 | 217 | **181** | **1413** | 1286 | 1523 |
| **EOS** | **/μL** | 0.32 | 745 | 6 | 36 | **8** | **741** | 693 | 797 |  |  |  |  |  |  |  |  |  |  |  |  |  |  |  |  |  |  |  |  |  |
| **BASO** | **/μL** | 0.10 | 748 | 1 | 2 | **2** | **270** | 241 | 300 |  |  |  |  |  |  |  |  |  |  |  |  |  |  |  |  |  |  |  |  |  |

TP; total protein, ALB; albumin, BUN; blood urea nitrogen, CRE; creatinine, T-BIL; total bilirubin, D-BIL; direct bilirubin, Na; sodium, K; potassium, CL; chlorine, Ca; calcium, CRP; C-reactive protein, AST; aspartate aminotransferase, ALT; alanine aminotransferase, LDH; lactate dehydrogenase, ALP; alkaline phosphatase, CK; creatine kinase, WBC; white blood cell, RBC; red blood cell, HGB; hemoglobin, HCT; hematocrit, PLT; platelet, NEUT; neutrophil, LYMP; lymphocyte, MONO; monocyte, EOS; eosinophil, BASO; basophil, GA; gestational age, RI; reference interval, LL; lower limit of the RI, UL; upper limit of the RI, CI; confidential interval (90%) of LLs and ULs were estimated by the bootstrap method. The results were excluded data of LDH, AST and CK in the infants with P20-29; Respiratory and cardiovascular disorders specific to the perinatal period, CRP with P35-39; Infections specific to the perinatal period, and K and T-Bil with P50-61; Hemorrhagic and homological disorders specific to fetus and newborn, respectively. A multivariate iterative method called latent abnormal value exclusion (LAVE) was applied to nine analytes (TP, BUN, K, LDH, ALT, WBC, CRP, HGB and HCT) which deemed to be adversely affected by hemolysis and inflammation. By use of 3-level nested ANOVA, the influence of GA on test results was expressed in terms of standard deviation (SD) ratio (SDR), as SDR_GA_. SDR>=0.4 were used as a criteria for the need of partition by the factor.
